# Genome-wide identification of bacterial colonization and fitness determinants on the floating macrophyte, duckweed

**DOI:** 10.1038/s42003-022-03014-7

**Published:** 2022-01-19

**Authors:** Hidehiro Ishizawa, Masashi Kuroda, Daisuke Inoue, Michihiko Ike

**Affiliations:** 1 Division of Sustainable Energy and Environmental Engineering, Graduate School of Engineering, Osaka University, 2-1 Yamadaoka, Suita, Osaka 565-0871 Japan; 2 Research Institute of Green Science and Technology, Shizuoka University, 3-5-1 Johoku, Naka-ku, Hamamatsu, Shizuoka, 432-8561 Japan; 3 Faculty of Social and Environmental Studies, Tokoha University, 6-1 Yayoi-cho, Suruga-Ku, Shizuoka, Shizuoka, 422-8581 Japan

**Keywords:** Water microbiology, Applied microbiology

## Abstract

Bacterial communities associated with aquatic macrophytes largely influence host primary production and nutrient cycling in freshwater environments; however, little is known about how specific bacteria migrate to and proliferate at this unique habitat. Here, we separately identified bacterial genes involved in the initial colonization and overall fitness on plant surface, using the genome-wide transposon sequencing (Tn-seq) of *Aquitalea magnusonii* H3, a plant growth-promoting bacterium of the floating macrophyte, duckweed. Functional annotation of identified genes indicated that initial colonization efficiency might be simply explained by motility and cell surface structure, while overall fitness was associated with diverse metabolic and regulatory functions. Genes involved in lipopolysaccharides and type-IV pili biosynthesis showed different contributions to colonization and fitness, reflecting their metabolic cost and profound roles in host association. These results provide a comprehensive genetic perspective on aquatic-plant-bacterial interactions, and highlight the potential trade-off between bacterial colonization and proliferation abilities on plant surface.

## Abstract (web summary)

Genome-wide transposon sequencing reveals the genes driving bacterial colonization of duckweed. These genes indicate a trade-off between the colonization and proliferation abilities on the host plant.

## Introduction

Macrophytes are key players in primary production and nutrient cycling in aquatic ecosystems, in which they interact with specific bacterial communities assembled from surrounding water environments^1–4^. The roles of these bacteria have long been emphasized in phytoremediation systems, in which bacterial denitrification and organic degradation account for a large part of water treatment performance^5–7^. Several plant growth-promoting bacteria have recently been isolated from aquatic energy crops such as duckweed; these bacteria offer possibility of accelerating biomass production from unutilized water environments^8–10^.

To date, however, the mechanistic aspects of bacterial assembly on macrophytes have yet to be studied in detail beyond 16 S rRNA gene profiling. In recent decades, studies utilizing model terrestrial plants and microbes (e.g., *Arabidopsis*-*Pseudomonas* interactions) have yielded considerable data on the bacterial genes involved in the establishment of plant-bacterial interactions^11,12^. Nevertheless, in terms of distinct biotic and abiotic conditions in terrestrial and aquatic environments, the functional involvement of these genes in aquatic-plant-bacterial interactions remains elusive. For example, larger ranges of bacterial movement and plant exudate diffusion in the aqueous phase are likely to alter the roles of fundamental bacterial functions such as motility and chemotaxis (c.f., bacteria can move up to ~2 cm d^–1^ through chemotaxis in soils^13^).

In the present study, we analyzed the bacterial gene functions involved in the establishment of aquatic-plant-bacterial interactions. To this end, we adopted a transposon mutagenesis method coupled with high-throughput insertion-site sequencing (Tn-seq^14^) because this technique can provide a genome-scale perspective of important genes, which is currently lacking for aquatic-plant-associated bacteria. Specifically, the interaction between a plant growth-promoting bacterium, *Aquitalea magnusonii* H3, and its original host, duckweed (*Lemna minor*), was analyzed. Due to its robust colonization and plant growth-promoting abilities, strain H3 has previously been utilized to investigate bacterial colonization and competition dynamics on the surface of duckweed^9,15,16^. Similar to other bacterial strains studied at the same time (*Acinetobacter* and *Asticcacaulis* strains), strain H3 can establish constant coexistence with duckweed, through rapid surface colonization and utilization of host-derived substrates^15^, while potentially inducing the host immune response^17^. More broadly, members of the genus *Aquitalea* (*Neisseriaceae*) are commonly found in freshwater environments at low abundance and associated with diverse macrophytes at specific population densities^18–21^. Thus, we considered strain H3 would be a suitable model strain with which to study in-depth aspects of aquatic-plant-bacterial interactions.

We also aimed to determine how each gene function of strain H3 contributes to the initial colonization and subsequent proliferation on duckweed’s surface. To thrive on plants, bacteria must first colonize the plants from surrounding environments, and subsequently, proliferate on plant surfaces more effectively than their competitors do. Although the genetic requirements for these two processes have not been well-distinguished in recent analytical approaches, the genes required for the first process might not always contribute to the latter process (and vice versa). Cell surface components such as flagella, lipopolysaccharides (LPS), and type-IV pili are considered typical colonization factors^22,23^. However, the large metabolic cost and host immune recognition associated with these components could negatively affect proliferation once bacteria have colonized. Peyraud et al.^24^ recently demonstrated the significant resource allocation trade-off between proliferation and production of costly virulence factors (e.g., extracellular polysaccharides) in the plant pathogen *Ralstonia solanacearum*. Improving the current understanding of these aspects will provide useful insights into the adaptive strategies of plant-associated bacteria, while potentially improving the delivery and survivability of beneficial bacteria to plant surfaces.

Herein, we report the identification of *A. magnusonii* H3 genes involved in the establishment of its association with duckweed. Based on a screening of transposon mutants with different experimental periods (3 h and 7 d), we separately identified the genetic requirements for colonization, i.e., migration and attachment to the plant surface, and fitness, i.e., overall population success resulting from both colonization and proliferation on the plant. Additional experiments were performed to ascertain the important roles of flagellar motility and chemotaxis in aquatic-plant-bacterial interactions.

## Results

### Tn-seq screening of colonization and fitness determinants

To screen bacterial genes involved in the establishment of aquatic-plant-bacterial interactions, we performed Tn-seq using *A. magnusonii* H3, a plant growth-promoting bacterium of duckweed *Lemna minor*. In Tn-seq, a saturated transposon mutant library was subjected to screening experiments that separate mutants with differing phenotypes^14,25^. Since the modified Himar1 transposon enables easy mapping of its insertion site *via* high-throughput sequencing, genome-wide screening can be performed in a single screening experiment.

Thus, we first created a mutant library of the spontaneous rifampicin-resistant mutant of *A. magnusonii* H3 (strain H3rifR) through the conjugative transfer of transposon vector pSAM_AraC^26^. Sequencing revealed that the mutant library had > 36,000 unique insertion sites (7.5 insertions per 1 kbp) distributed across the genome of strain H3 (Supplementary Fig. 1). Given the low mutation frequency (0.027%), mutants with multiple insertions would have been very rare. Of the 4443 protein-coding genes in the genome, 3966 (89.3%) had at least one insertion event (mean and median insertion sites per gene: 7.6 and 6, respectively). Among 477 genes without detectable mutants, 299 (62.7%) showed significant similarity to genes in the Database of Essential Genes^27^ (*e*-value < 0.001; Supplementary Data 1), suggesting that our mutant library provides a near-exhaustive screening of nonlethal genes.

**Fig. 1:**
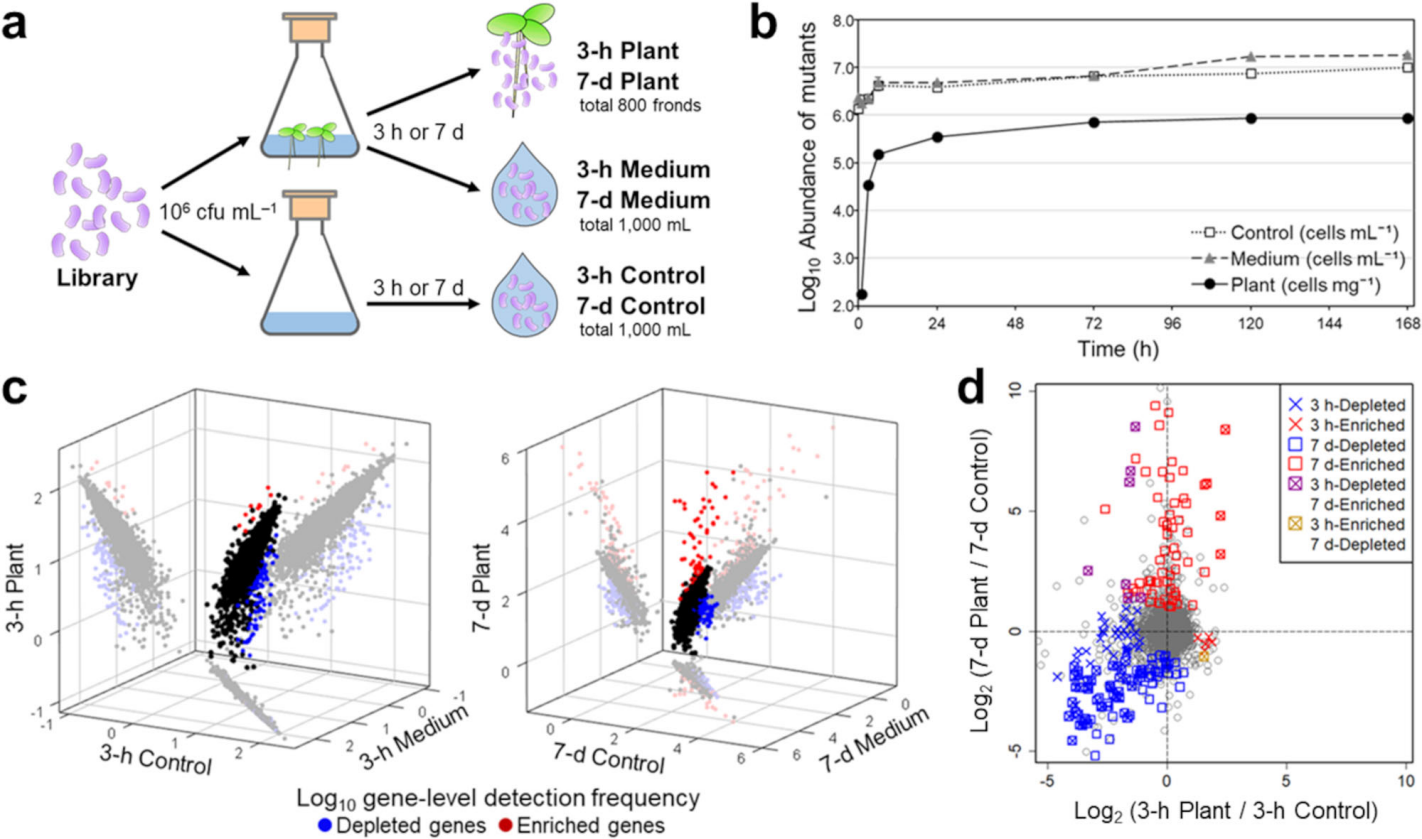
Tn-seq screening identified colonization and fitness determinants. **a** Schematic diagram of the screening experiments. The transposon mutant library of *Aquitalea magnusonii* H3rifR was inoculated to flasks with and without duckweed, and the DNA of mutants was collected from three fractions after 3 h or 7 d. Samples were destructively obtained from four flasks, and pooled before DNA extraction. **b** The abundance of mutant cells in the three fractions during the screening experiments. Error bars show standard deviation. **c** Detection frequency of genes in the three fractions after 3 h and 7 d. Log_10_ gene-level detection frequencies were compared. Depleted and enriched genes were determined based on the effect size (fold change > 2) and statistical significance (*q* < 0.05) among Plant and Control samples. Statistical test was performed by considering different mutants (insertion sites) of the same gene as replicates. **d** Comparison of the screening results of 3-h and 7-d experiments. Genes showed significant depletion or enrichment in the 3-h and 7-d experiments are denoted by crosses and squares, respectively.

The mutant library was inoculated to modified Hoagland medium (inorganic medium) with and without *L. minor* plants (Fig. 1a). After incubations of 3 h or 7 d, samples were destructively collected from three fractions; Plant (adhered to duckweed after harvest and gentle wiping with a paper towel), Medium (culture medium with duckweed), and Control (culture medium without duckweed). The samples obtained from four flasks were combined, and the DNA of mutant cells was extracted for further analyses. Quantification of mutant cells in each compartment by qPCR indicated that strain H3 mutants rapidly adhered to the plants within 6 h and maintained their full colonization density for a long period (Fig. 1b), consistent with previous observations for wild-type strain H3^15^. The total amount of cells in a culture flask (Plant + Medium) did not change remarkably until after 6 h; a ~10-fold increase relative to the original inoculated amount was observed at 7 d. Thus, the mutant composition in Plant samples after 3 h reflects the pure colonization ability of mutants, i.e., the influence of cell growth and mortality is limited at this stage. On the other hand, the results at 7 d reflect the overall bacterial fitness while on the plant surface, including the efficiencies of both colonization and proliferation.

When the transposon-flanking region of the DNA samples was amplified and sequenced, we obtained ~17.3–24.0 million reads for each sample, which were successfully mapped to the genome of strain H3 (on average 567 reads per insertion site). Comparisons of gene-level detection frequencies among samples indicated that results for 3-h Control and 3-h Medium were similar (Pearson’s *r* = 0.999; Fig. 1c) and almost unchanged from those of the inoculated mutant library (*r* = 0.991; Supplementary Fig. 2). On the other hand, 3-h Plant showed different compositions to 3-h Control and 3-h Medium (*r* = 0.906–0.908), reflecting the variation in colonization efficiency among mutants. Likewise, 7-d Control and 7-d Medium were highly correlated (*r* = 0.977) whereas 7-d Plant exhibited a different mutation profile (*r* = 0.798–0.886). Therefore, the presence of duckweed plants seemed to have a minor influence on mutant composition in the liquid phase and strong selection appears to have occurred at the plant-water interface.

Thus, for each of the 3-h and 7-d experiments, we compared the detection frequencies in Plant and Control samples to determine genes whose mutants were significantly depleted or enriched in Plant samples. A statistical test (two-sided *t*-test) was performed for 3373–3401 genes with multiple (2–96) insertion sites, by considering different mutants of the same gene as replicates. Then, highly significant genes satisfying false discovery rate (FDR)-corrected *p*-value (*q*-value) < 0.05 (i.e., the proportion of incorrectly detected genes was kept below 5%) and Log_2_ fold change in detection frequencies (Plant/Control) < –1 or >1 were determined as depleted or enriched genes, respectively (Supplementary Fig. 3; Materials and Methods). Here, Log_2_ fold change values are considered to represent the colonization or fitness ability of mutants; hence, depleted and enriched genes are deemed to have beneficial and detrimental roles, respectively, in the colonization or fitness^28^. Through this procedure, we identified 86 and ten depleted and enriched genes, respectively, in the 3-h experiment; in the 7-d experiment, we identified a similar number of depleted genes (83 genes) but many more enriched genes (65 genes) (Supplementary Data 2-6). Although the identification results for depleted genes had certain similarity between the 3-h and 7-d experiments, results for enriched genes were hardly relevant (Fig. 1d).

Altogether, the Tn-seq screening identified different sets of strain H3 genes that likely play important roles in the initial colonization and overall fitness on the surface of duckweed. A possible bias is that genes with a few insertion sites (likely small genes) are less probable to be detected, given the statistical power to detect depleted/enriched genes increases with the number of unique insertion sites per gene (Supplementary Fig. 4).

### General functions of identified genes

**Fig. 2:**
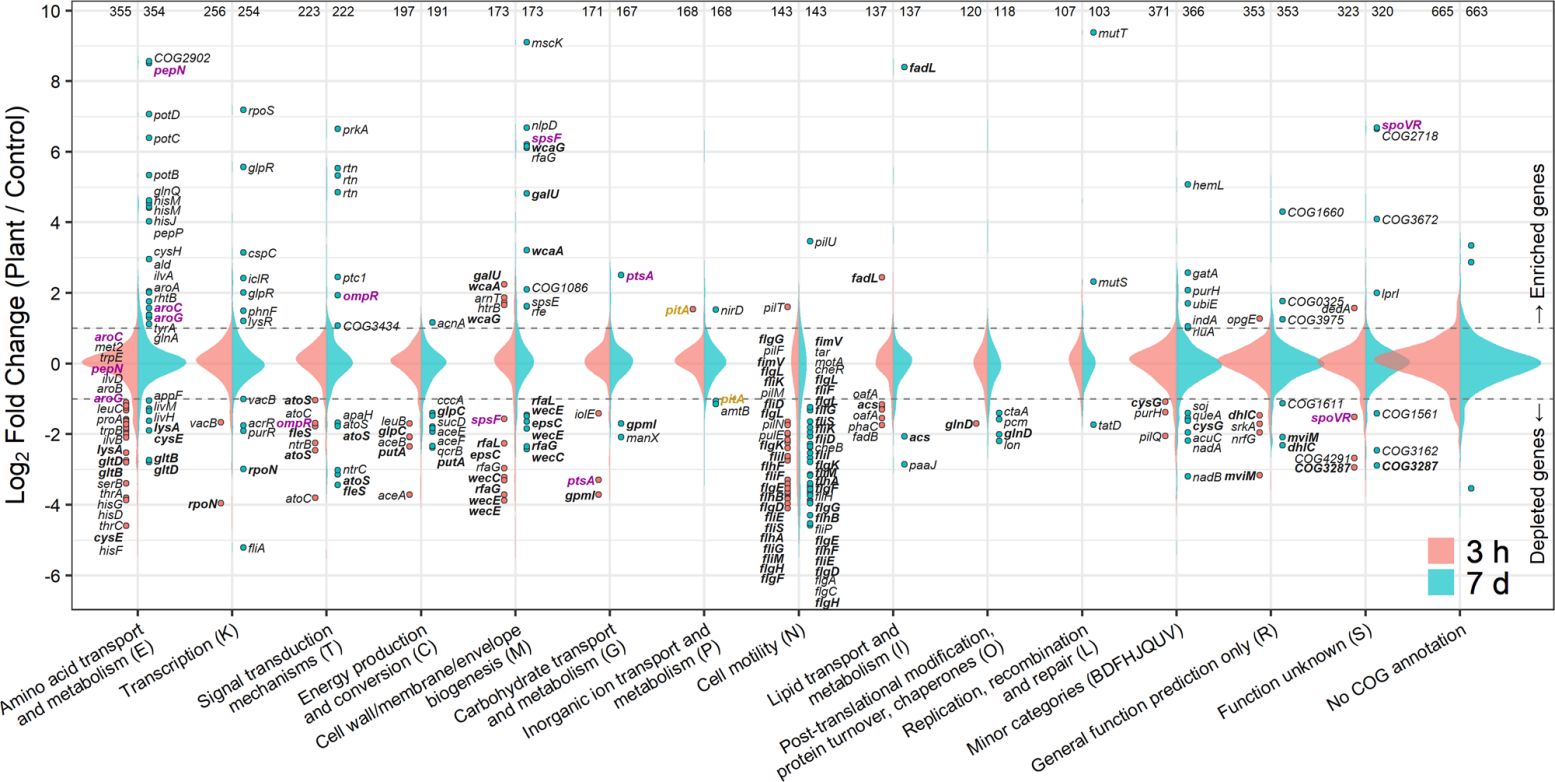
Distribution of the colonization/fitness ability of transposon mutants. Results are shown according to functional categories based on the COG database. Depleted genes (Log_2_ Plant/Control < –1; *q* < 0.05) and enriched genes (Log_2_ Plant/Control > 1; *q* < 0.05) are independently plotted. Genes that were consistently depleted or enriched across time points are shown in bold, and those with opposite results are shown in purple or yellow. Values at the top indicate the number of tested genes in each category.

In Fig. 2, we summarize the screening results based on the functional categories of the COG database^29^. Irrespective of functional classifications, the majority of genes were neutral for colonization and fitness (i.e., Log_2_ fold change = ~0). However, the Cell motility, Cell wall/membrane/envelope biogenesis, and Amino acid transport and metabolism categories contained a significantly large number of depleted or enriched genes (*p* < 0.01, Fisher’s exact test; Supplementary Table 1). The differences between 3-h and 7-d experiments were substantial for Amino acid transport and metabolism, Transcription, Signal transduction mechanism, and Cell wall/membrane/envelope biogenesis, in which many and/or strong enriched genes were specifically found in the 7-d experiment (Fig. 2). Further comparison of genetic contribution across time points is shown in Supplementary Fig. 5.

### Specific functional roles of identified genes

**Fig. 3:**
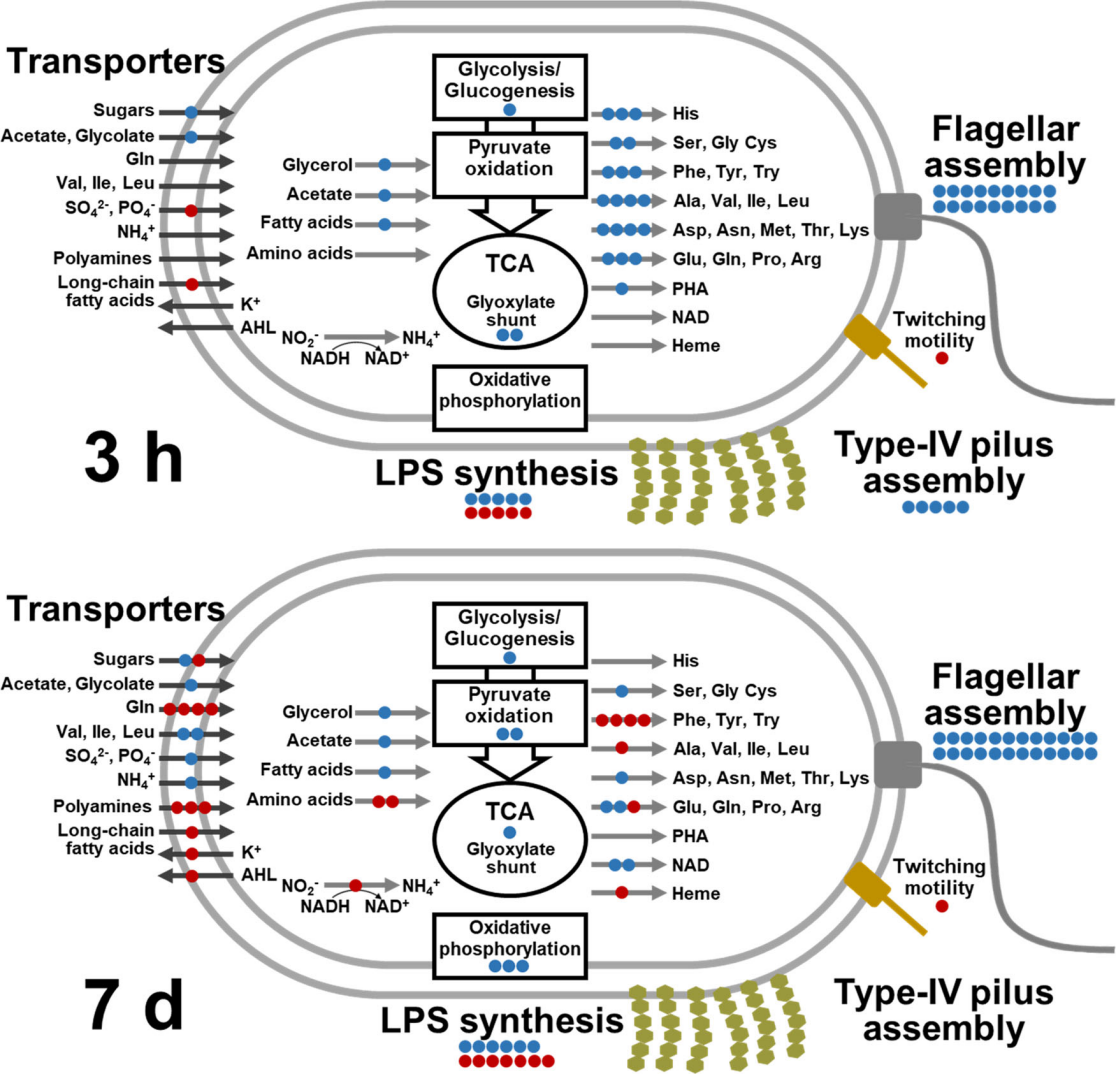
Important metabolic pathways and cellular components for the efficient duckweed colonization (3 h) and fitness (7 d) of *Aquitalea magnusonii* H3. Blue and red circles represent the occurrence of depleted and enriched genes, respectively, in the corresponding pathways or functions. Gene roles were predicted according to the best-matched entry in the KEGG orthology database. Amino acids are denoted by their standard three-letter code. LPS, lipopolysaccharide; PHA, polyhydroxyalkanoate; AHL, acyl-homoserine lactone. Supplementary Data 8 shows the list of depleted and enriched genes in this figure.

We further predicted the detailed roles of the depleted and enriched genes based on the best-matched functional orthologs (*k* numbers) from the KEGG database^30^. As summarized in Fig. 3, genes for flagellar assembly showed obvious depletion in both 3-h and 7-d experiments. Genes involved in LPS and spore-coat polysaccharide biosynthesis (e.g., *wecC*, *wecE*, *rfaG*, *rfaL*, *galU*, *wcaA*, *wcaG*, *mviM*, *spsE*, and *spsF*; Supplementary Data 2) were either depleted or enriched, probably reflecting their profound roles in plant attachment and host immune response^31,32^. Notably, these depleted and enriched genes showed larger Log_2_ fold change values after 7 d than after 3 h without any exception (Fig. 2; Supplementary Fig. 5). This suggests that these cell surface polysaccharides consistently antagonize post-colonization proliferation on plant surface. Similarly, genes encoding type-IV pili were significantly depleted after 3 h but not after 7 d. Genes involved in twitching motility *pilT* (DLM_4021) and *pilU* (DLM_4022) were enriched after 3 h and 7 d.

In terms of metabolic function, many biosynthetic genes for proteogenic amino acids were significantly depleted after 3 h, whereas many were no longer significant after 7 d. In contrast, biosynthetic genes for aromatic amino acids were enriched after 7 d. In the 7-d experiment, a higher level of depleted genes was identified in central metabolic pathways, oxidative phosphorylation, and NAD biosynthesis, suggesting the importance of efficient energy metabolism for bacterial fitness. Genes for utilization of glycerol, acetate, and fatty acids also appeared to be important, which is further supported by findings for relevant transcriptional regulators and membrane transporters (*glpR*, DLM_3899, and DLM_4133; *dhlC*, DLM_0668).

In the 7-d experiment, many depleted or enriched genes encoded membrane transporters. Among these genes, those involved in the intake of probable carbon and nitrogen sources (e.g., sugars, amino acids, and ammonium) showed weak (fold change < 3) but significant depletion. In addition, genes encoding long-chain fatty acid transporter (*fadL*, DLM_1061) and polyamines (*potBCD*, DLM_1951–1953) were exceptionally enriched by ~100-fold (Fig. 2). Probable efflux transporter of acyl-homoserine lactone (*rhtB*, DLM_0096) was also significantly enriched after 7 d.

The 7-d experiment also revealed many enriched genes for which roles in bacterial fitness could not be readily inferred, e.g., aminopeptidase genes (*pepN*, DLM_0384; *pepP*, DLM_3891) and DNA repair genes (*mutS*, DLM_3294; *mutT*, DLM_0823). The significant influences of genes contributing to osmosensing (*ompR*, DLM_0023), K^+^ efflux (*mscK*, DLM_0821), and peptidoglycan integrity (*nlpD*, DLM_3315; *mviM*, DLM_4446) may indicate the importance of osmotic adaptation, similar to those reported for terrestrial rhizobacteria^33^.

### Regulatory genes for flagellar motility suggested the merit of high-motile phenotype

**Fig. 4:**
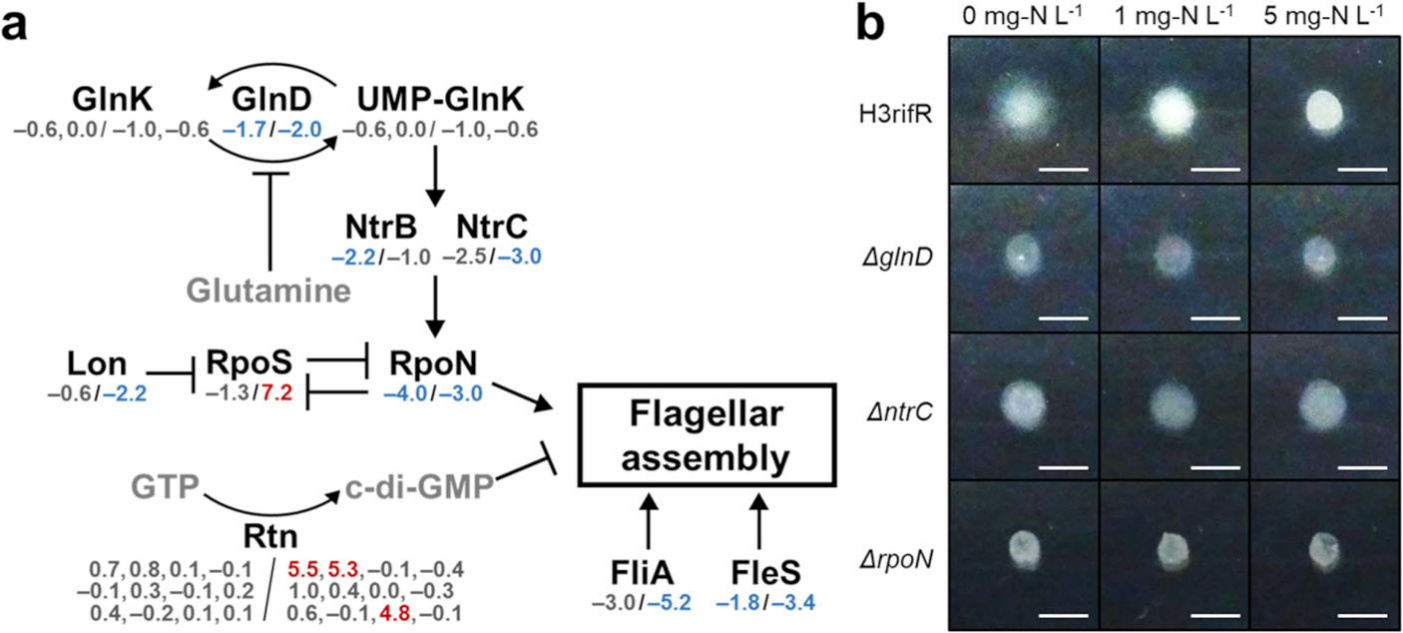
Signaling genes for flagellar motility are critical for efficient colonization and fitness. **a** Predicted signaling network for flagellar assembly of *Aquitalea magnusonii* H3. Values below protein names represent the effects on colonization/fitness ability (Log_2_ Plant/Control; values before and after slash represent the results after 3-h and 7-d experiments, respectively; Fig. 2) of corresponding genes. Depleted and enriched genes are shown in blue and red, respectively **b** The hypermotile phenotype of strain H3rifR (parental strain) under nitrogen starvation and the loss of this response upon mutation in the signal cascade. The cell suspension was spotted on a soft agar plate with different nitrogen concentrations, and colony expansion was observed after 8 h. Scale bar represents 5 mm. Supplementary Data 9 shows the list of depleted and enriched genes in this figure.

Many regulatory genes for flagellar assembly showed strong depletion or enrichment, in the direction to support the importance of flagellar motility (Fig. 4a). For example, *glnD* (DLM_2298), *ntrB* (DLM_3083)*, ntrC* (DLM_3084), and *rpoN* (DLM_0255) belong to the same signal cascade that senses cellular glutamine levels and promotes flagellar assembly under nitrogen starvation^34,35^. Indeed, the hypermotile phenotype of strain H3rifR was observed under low nitrogen conditions, whereas such a response was abolished upon the deletion of *glnD, ntrC*, and *rpoN* (Fig. 4b). Genes responsible for c-di-GMP signaling (*rtn*, DLM_0332, DLM_1146, and DLM_3694), which plays a central role in the switch from a motile to sessile lifestyle in diverse bacteria^36^, were strongly enriched after 7 d.

### The role of chemotaxis in plant colonization and fitness

**Fig. 5:**
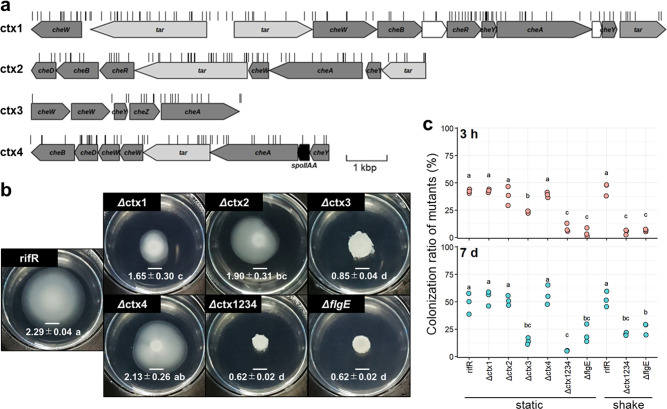
Contribution of chemotaxis to colonization and fitness. **a** Gene maps of four chemotaxis gene clusters in the strain H3 genome. Depleted or enriched genes were not found in the four-gene clusters (Supplementary Data 7). **b** Chemotactic motility of the parental strain (rifR), mutants lacking each and all four chemotaxis gene clusters (*Δ*ctx1, *Δ*ctx2, *Δ*ctx3, *Δ*ctx4, and *Δ*ctx1234), and nonmotile mutant (*ΔflgE*) on a soft agar plate. The average diameter (cm ± SD) of colonies is shown. Scale bar represents 5 mm. **c** Results of co-inoculation assays against the wild-type strain under static and shaken conditions. Plots represent the ratio of mutant cells colonized on duckweed. Different lowercase letters denote significant differences (*P* < 0.05; Tukey’s HSD test).

Despite the apparent importance of flagellar motility, no genes in the four chemotaxis gene clusters of the strain H3 genome showed a significant influence (Fig. 5a; Supplementary Data 7). This suggests that chemotactic navigation might not be needed for duckweed colonization in the aqueous phase. Alternatively, Tn-seq may have failed to abolish the chemotactic ability of strain H3 due to genetic redundancy. To examine these possibilities, we constructed mutants lacking each and all of the four chemotaxis gene clusters (*Δ*ctx1234, *Δ*ctx1, *Δ*ctx2, *Δ*ctx3, and *Δ*ctx4). Microscopy observations confirmed that all of the mutants retained swimming motility, while all but *Δ*ctx4 exhibited impaired chemotactic motility in the soft agar assay (Fig. 5b).

To evaluate the competitive ability of the mutants, each chemotaxis mutant and nonmotile mutant (*ΔflgE*, DLM_2444) was inoculated to duckweed as a 1:1 mixture with the wild-type strain; the proportion of mutants attached on the plants was then quantified using selective agar plates. As a result, deletion of all chemotaxis gene clusters (*Δ*ctx1234) severely reduced competitive ability to a level similar to that of nonmotile mutants (*ΔflgE*) (Fig. 5c). On the other hand, mutation of single gene clusters had a weaker influence than *Δ*ctx1234, suggesting that the four-gene clusters have complementary functions. Collectively, the non-discovery of chemotaxis-related genes in the Tn-seq could be attributed to genetic redundancy; chemotaxis plays a critical role in colonization and fitness.

Since the presence of a water current in aquatic environments is likely to affect the contribution of chemotaxis and flagellar motility, we further tested the competitive abilities of *Δ*ctx1234 and *ΔflgE* mutants under conditions in which culture flasks were shaken at 100 rpm throughout the cultivation term. Results consistently showed the weaker competitive abilities of mutants (Fig. 5c), which supports the contribution of chemotaxis and flagellar motility in the presence of a water current.

### Validation of the roles of beneficial/detrimental genes identified by Tn-seq

**Fig. 6:**
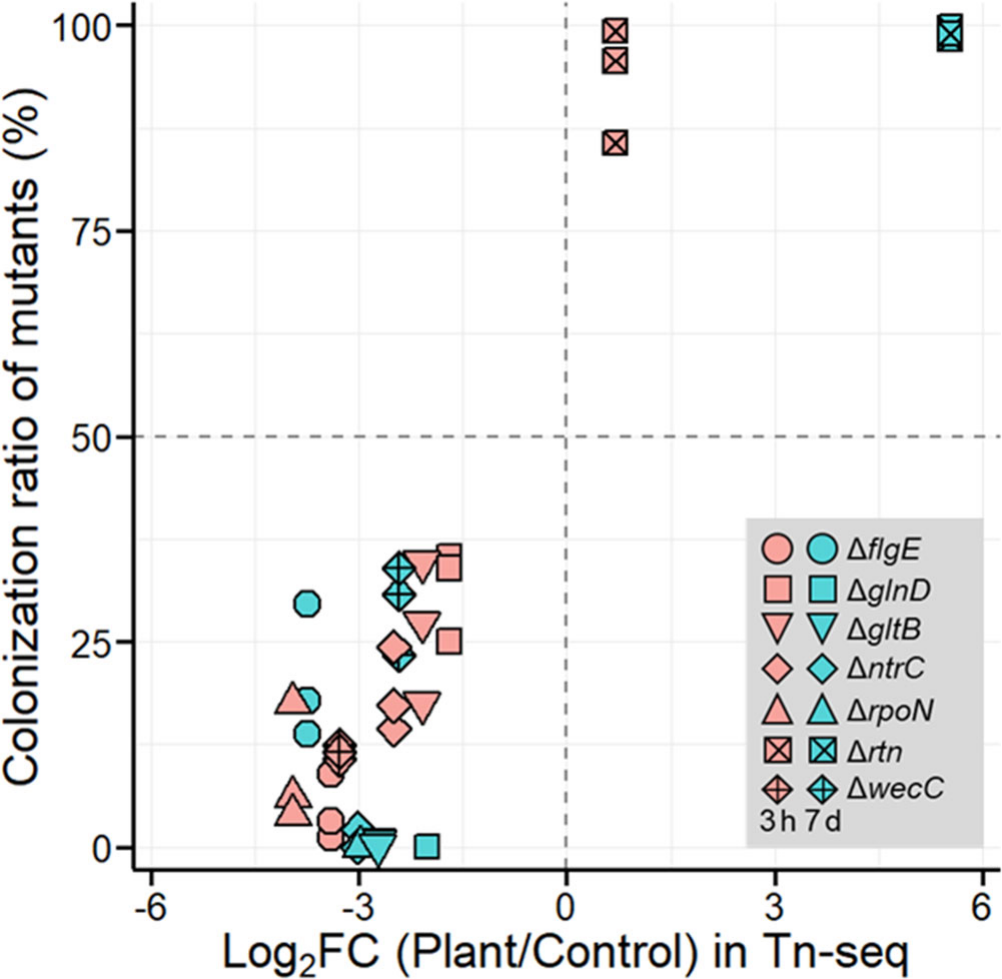
Validation of Tn-seq results by the co-inoculation assay. Seven deletion mutants (shown in Table 1) were subjected to the co-inoculation assay with the wild-type strain; the colonization ratio of mutants was plotted against the results of corresponding genes in the Tn-seq.

To examine if the genes identified by the Tn-seq actually exhibit different colonization/fitness abilities, we constructed deletion mutants of seven depleted/enriched genes representing diverse cellular and metabolic functions (Table 1), and evaluated their colonization/fitness abilities by co-inoculation assay with the wild type. The results showed that depletion and enrichment results in the Tn-seq were successfully reproduced, as corresponding mutants showed a significantly larger or smaller colonization ratio than the wild type (two-sided *t*-test; *p* < 0.05) (Fig. 6). The minor differences were that the significant effects of *ntrC* and *rtn* after 3 h were confirmed by the co-inoculation assay but not by the Tn-seq. The change in colonization/fitness abilities cannot be solely due to the change in growth rate because the specific growth rates of mutants were similar to those of the wild-type strain (0.79 ± 0.01 h^−1^; Table 1). These results support the reliability of colonization or fitness determinants identified through our Tn-seq screening.

**Table 1:** Genes selected for the validation experiments.

Locus tag	Gene name	Predicted function	Log_2_ (Plant/Control)		Specific growth rate in LB (h^−1^ ± SD)
			3 h	7 d	
DLM_2444	*flgE*	Flagellar hook protein	−3.4***	−3.7***	0.87 ± 0.02
DLM_2298	*glnD*	Protein PII uridylyltransferase	−1.7**	−2.0**	0.62 ± 0.03
DLM_3387	*gltB*	Glutamate synthase large chain	−2.1***	−2.7***	0.79 ± 0.04
DLM_3084	*ntrC*	Nitrogen regulation protein	−2.5	−3.0*	0.81 ± 0.00
DLM_0255	*rpoN*	RNA polymerase sigma-54 factor	−4.0***	−3.0***	0.78 ± 0.03
DLM_0332	*rtn*	Diguanylate cyclase/phosphodiesterase	0.6	5.5***	0.81 ± 0.00
DLM_4445	*wecC*	UDP-N-acetyl-D-mannosaminuronate dehydrogenase	−3.3***	−2.4**	0.84 ± 0.00

The results of Tn-seq screening and the specific growth rate of corresponding mutants are shown.

*q < 0.05; **q < 0.01; ***q < 0.001.

## Discussion

In this study, we reported the genome-wide identification of *A. magnusonii* H3 genes that contribute to its association with duckweed, *L. minor*. The majority of identified genes encoded products with closely related functions and the follow-up experiments showed a high validation rate of the screening results. Although several relevant genes would have been left undetected due to the limited statistical power, the dataset would be robust in identifying important cellular functions and metabolic pathways. One consideration is that our screening failed to clarify the importance of bacterial chemotaxis. Since the Tn-seq approach relies on single-gene mutations, careful interpretation is necessary for functions encoded by redundant genes. For example, our Tn-seq identified only a few genes involved in the central metabolic pathway and chemotaxis receptors which are abundant in the strain H3 genome; these might also be due to genetic redundancy.

Evaluation of 3 h and 7 d periods in this study highlighted the distinct genetic requirements for initial plant colonization and overall fitness. For initial colonization, more than half of the identified genes encoded flagella, LPS, type-IV pili, or relevant regulatory proteins. In addition, chemotaxis was found to have critical roles even under the presence of water current. These results agree with the conventional view that such cellular components are the most direct participants in bacterial attachment to plant surfaces^24^, even in aquatic context. Although the 3-h experiment would be insensitive to differences in mutant growth rate, several genes related to carbon utilization and amino acid biosynthesis were found to be beneficial. Rather than directly impairing colonization ability, the mutation of these genes likely perturbed normal cellular functions at the beginning of the screening experiments and thereby delayed colonization on the plant.

Interestingly, the contributions of genes related to LPS, spore-coat polysaccharides, and type-IV pili consistently showed larger Log_2_ fold change value after 7 d than 3 h. This suggests that these cell surface components may become a metabolic burden or unfavorable host immune elicitor once attached to plant surface, despite their importance in the colonization process. Similarly, the negative effects of aromatic amino acid biosynthesis on fitness suggest the benefit of amino acid auxotrophy in the plant environment^28^ given the especially high biosynthetic cost of aromatic amino acids^37^. Altogether, it was suggested that some gene functions required for efficient colonization might significantly compromise proliferation on the plant surface, probably due to resource allocation trade-off^24^ or other mechanisms.

On the other hand, our results related to regulatory genes consistently indicated that flagellar motility of strain H3 play essential roles even after colonization, unlike many terrestrial plant-associated bacteria switch from motile to sessile (or biofilm-forming) lifestyles upon colonization^38^. This is compatible with the previous observation that strain H3 colonizes duckweed surfaces as dispersed microcolonies of 1–10 cells^15^, which is an indication of frequent dispersal and recolonization. Since gathering at high density would compromise the growth rate of individual cells and more likely trigger host immune defense, high-dispersal phenotypes of plant-associated bacteria sometimes shows superior fitness to that of biofilm-forming phenotypes^39,40^. Thus, we speculate that quicker cycles of colonization, growth, and dispersal at the plant-water interface eventually improve the overall fitness of strain H3. In previous research, microscopy of naturally grown duckweed surfaces has observed dispersed bacterial cells rather than thick biofilms^1,41^. These results indicate that the dispersal strategy is likely widespread in aquatic-plant-bacterial interactions, where bacteria can disperse more easily than they do in soils.

Many more enriched genes were found in the results of fitness determinants than were found in the results of colonization determinants. We infer that unfavorable cell surface structures (e.g., LPS-related genes) and unfavorable transcriptional regulations (e.g., genes repressing flagellar motility) were the major mechanisms that severely suppressed the fitness of strain H3. In addition, transporters for long-chain fatty acid and polyamines had a highly negative impact. Although such strong influence was unexpected for genes participating only in substrate uptake, long-chain fatty acid transporter *fadL* could be responsible for the intake of acyl-homoserine lactone, a major quorum-sensing molecule of gram-negative bacteria^42^. Polyamines are also known to be signaling molecules secreted by plants and bacteria^40,43,44^ and their uptake is reported to decrease the rhizosphere fitness of *Pseudomonas fluorescens*^45^. Hence, these membrane transporters might be involved in the intercellular communications that restrict the fitness of strain H3.

In summary, using strain H3 as a model, this study provides a comprehensive genetic perspective on the establishment of aquatic-plant-bacterial interactions. Initial colonization efficiency seems to be governed mainly by flagellar motility, chemotaxis, and cell surface structures, whereas diverse gene functions positively and negatively regulate overall fitness on the plant surface. Flagellar motility and its regulatory mechanisms were revealed as the most critical functions in both colonization and fitness, implying that the modulation of dispersal and recolonization represents the key adaptive strategy at the plant-water interface. Furthermore, the production of some colonization factors, such as LPS and type-IV pili, was suggested to compromise bacterial proliferation on plant surface, highlighting the potential trade-off between bacterial colonization and fitness abilities. Finally, our work provides the molecular basis for manipulating aquatic-plant-bacterial interactions toward the development of highly efficient plant hydroculture techniques employing beneficial bacteria.

## Methods

### Plants, bacterial strains, and growth media

Laboratory stock of sterile *L. minor* (#RDSC5512) was used in this study. The plants were sterilized with sodium hypochlorite^46^ and successively cultured in clonal multiplication mode with modified Hoagland medium^47^ under standard laboratory conditions (28 °C, photon flux of 80 µmol m^−2^ s^−1^, and a 16/8-h light/dark cycle). Plants used in experiments were randomly picked from pre-cultivation flasks that had been incubated for 10 d or less.

The bacterial strains, vectors, and primers used in this study are summarized in Supplementary Data 10. LB (Lennox) medium was used to grow *A. magnusonii* H3, its spontaneous rifampicin-resistant mutant (H3rifR), and *Escherichia coli* strains. When necessary, 10 µg mL^−1^ rifampicin (rif), 100 µg mL^−1^ ampicillin (amp), 50 µg mL^−1^ kanamycin (kan), 10 mM D-glucose (glc), and 5% sucrose (suc) was added to the media. 5-bromo-4-chloro-3-indolyl-β-D-galactoside (X-Gal; 40 µg mL^−1^) was used to detect *E. coli* strains that inadvertently occurred on the selective media.

### Construction of the transposon mutant library

The mutant library of H3rifR was created with transposon vector pSAM_AraC^26^ (Addgene plasmid #91569). The donor strain (*E. coli* S17-1λpir/pSAM_AraC) and recipient strain (H3rifR) were grown overnight in 20 mL of LB + rif + kan + glc and LB + rif media, respectively, and then washed twice with LB medium. Subsequently, the cells were mixed at a donor-to-recipient ratio of ~4:1 in 100 µL of 1-M arabinose solution at an OD_600_ of ~1.0, before being inoculated to 0.45 µm nitrocellulose filter disks (Merck Millipore, Darmstadt, Germany) on LB agar. After incubation at 28 °C for 2 h, the cells on the filter disks were resuspended in fresh LB medium and the diluents were spread on LB + rif + kan + X-Gal agar plates to obtain insertion mutants. The mutation frequency was calculated based on a comparison of colony counts with those from non-selective (LB + rif + X-Gal) plates, and successful insertion was verified *via* a PCR. After incubation at 28 °C for 36 h, the mutant colonies that occurred on plates (~200 colonies per plate) were resuspended in LB with a final OD_600_ of ~3.0. Approximately 10,000 mutant colonies were pooled in each of five independent mutagenesis procedures; thus, ~50,000 colonies were ultimately preserved with 15% glycerol at −80 °C.

### Screening experiments

Mutant library stock (3.5 mL) was added to 20 mL of LB + rif+kan medium, grown for 4 h (at 28 °C and 120 rpm), and washed twice with sterile modified Hoagland medium. The cells were inoculated to flasks containing 250 mL of modified Hoagland medium with and without *L. minor* at ~10^6^ cfu mL^−1^, which corresponds to an average of ~7000 cells per mutant in each flask (Fig. 1a). After incubation in the growth chamber for 3 h or 7 d, 200 fronds of duckweed were collected, gently wiped with sterile paper towels, and preserved at −80 °C as Plant samples. To collect Control and Medium samples, cells in the liquid phase were collected on a 0.2 µm pore-sized filter and preserved at −80 °C. Samples were obtained from each of the four flasks and combined before DNA extraction. Parts of the mutant library inoculated to the flasks were preserved as Library samples.

### Tn-seq library preparation and sequencing

Tn-seq library preparation and sequencing were performed according to Goodman et al.^48^. Briefly, DNA was isolated from the obtained samples with 300 µL of Cica Genius DNA Extraction Solution (Kanto Chemical, Tokyo, Japan) and the transposon-flanking region was amplified by a liner PCR using primer BioSamA (Supplementary Data 10). The PCR products were purified using a QIA Quick PCR Purification Kit (Qiagen, Hilden, Germany) and bound to Dynabeads M-280 Streptavidin (Invitrogen, Carlsbad, CA, USA) before double-stranded by Klenow (exo-) (New England Biolabs, Ipswich, MA, USA) and hexanucleotide mix (Sigma Aldrich, St. Louis, MO, USA). The DNA was then digested with MmeI (New England Biolabs), ligated with barcoded adapters, and amplified with primers LIB-PCR3 and LIB-PCR5 (Supplementary Data 10). Finally, PCR products with ~125 bp in size were sequenced on an Illumina Hiseq 2500 platform (Illumina, San Diego, CA, USA) in 50-bp single-read mode.

### Tn-seq data analysis

Sequence reads were quality filtered (> Q30) using prinseq v0.20.4 and mapped to the complete genome sequence of strain H3^49^ (coverage 198x; Genbank AP018823) using pyinseq v0.2.0 (https://github.com/mjmlab/pyinseq). The insertion site data were removed if the total read count was < 10. Using “binom” function of R v3.6.2, two-sided binomial tests were performed to eliminate insertion sites for which the read counts mapped to the right- and left-side sequences were unnaturally different (> 10-fold difference, *p* < 0.05). Read count data distributed in 36,175 insertion sites were then added with unity and divided by the median read count of each sample. Gene-level detection frequency was calculated as the sum of the median-normalized read counts in the same open reading frame.

Differences in gene-level detection frequencies among samples were tested as follows. First, for each insertion site in the same open reading frame, Log_2_ fold changes of median-normalized read counts were calculated among compared samples (e.g., 3-h Plant *vs*. 3-h Control). Only insertion sites with a total ≥ 30 actual read counts in compared samples were considered. Next, one-sample two-sided Student’s *t*-test was done to determine whether the mean Log_2_ fold-change values were significantly larger or smaller than zero. This process was completed for 3373–3401 genes with ≥ 2 valid insertion sites to yield *p*-values as much as tested genes, which were then converted into *q*-values using the “qvalue” package in R. Finally, genes satisfying *q* < 0.05, corresponding to an FDR of 5%, and showing a Log_2_ fold change in gene-level detection frequency of < –1 or > 1 were deemed significant.

A homology search against the COG database and Database of Essential Genes^27^ were performed with rpsblast^50^. Fisher’s exact test was used to identify COG categories containing an enriched number of beneficial and detrimental genes. Detailed gene functions and pathways were predicted based on annotations in the KEGG database, using Blast KOALA, Ghost KOALA, and KAAS^51,52^.

### qPCR

Samples for SYBR Green qPCR analyses were collected from the same experimental setup used in the screening experiments. From triplicate flasks with and without *L. minor*, Plant, Medium, and Control samples were collected after 1 h, 3 h, 6 h, 1 d, 3 d, 5 d, and 7 d; DNA was then extracted as described above. The qPCR was performed using primers H3f and H3r (Supplementary Data 10) with GeneAce SYBR qPCR Mix α (Nippon Gene, Tokyo, Japan) in the CFX Connect Real-Time PCR Detection System (Bio-Rad, Hercules, CA, USA) platform.

### Construction of mutants by homologous recombination

Targeted mutagenesis was completed by double-crossover homologous recombination. For each targeted gene or gene cluster, ~1 kbp regions of upstream and downstream sequences were amplified by PCR (Supplementary Data 10) and then cloned into pK18mobsacB^53^ using In-Fusion HD Cloning Kit (Takara Bio, Tokyo, Japan). The constructs were introduced by mating on a 0.45 µm filter disk on LB agar, with an ~1:1:1 mixture of recipient strain (H3rifR or its derivative), donor strain (*E. coli* DH5α/pK18mobsacB construct), and helper strain (*E. coli* DH5α/pRK2013). After incubation at 28 °C for 15 h, transformants were obtained on an LB + rif + kan agar plate. The second crossover was completed by spreading a log culture of the transformants on an LB + rif + suc plate. The specific growth rates of mutants were evaluated from growth curves in 5 mL LB medium (*n* = 3) recorded with a biophotorecorder (TVS062CA; Advantec, Tokyo, Japan).

### Co-inoculation assay

Log cultures of the mutant and wild-type cells were washed twice, mixed at a 1:1 ratio, and inoculated to 60 mL of modified Hoagland medium in flasks at 10^6^ cells mL^−1^. Ten fronds of sterile *L. minor* were then added in each flask and cultivated in a growth chamber for 3 h and 7 d. For selected mutants (*ΔflgE* and *Δ*ctx1234), the incubation was also conducted with the flasks were shaken with orbital shaker at 100 rpm, which should produce a stronger water current than normal growing environments of duckweeds (e.g., ponds). To estimate the colonization density of wild-type and mutant cells, plants were gently wiped with sterile paper towels and homogenized using Biomasher II (Nippi, Tokyo, Japan) before the homogenates were spread on both 1/10 LB and 1/10 LB + rif plates. Since the total bacterial colonization density was constant (~10^6^ cfu mg^−1^), the competitive ability of mutants was estimated as the proportion of the rif-resistant colonies.

### Motility assay

To evaluate flagellar motility, 10 µL log cultures of strain H3rifR and mutants (OD_600_ = 1.0) were spotted on soft agar medium containing 0.25% agar, and colony expansion was recorded after 8-h incubations at 28 °C. The soft agar medium formulation was identical to that used as a plant culture medium in the screening experiments, except that 20-mM D-glucose was added as the carbon source and the concentration of KNO_3_ (original concentration: 5 mg-N L^−1^) was adjusted to 0, 1, and 5 mg-N L^−1^.

The chemotactic motility of mutants was evaluated using 1/10 LB soft agar medium with 0.25% agar supplemented with 0.1% casamino acid as chemoattractant. Log cultures of tested strains (OD_600_ = 1.0) were spotted on the center of soft agar, which were photographed after a 5 d incubation at 28 °C. The diameters of colonies were evaluated using ImageJ software v1.51n.

### Statistics and reproducibility

Statistical analyses were performed using R v3.6.2. Tn-seq data was processed as described in “Tn-seq data analysis” section. Three biological replicates (samples from different flasks) and every three technical replicates were used for qPCR quantification. Co-inoculation assay and motility assay were performed with three and six biological replicates, respectively, and difference among groups was tested by one-way ANOVA and Tukey’s post hoc tests. Significant difference of mutant competitive ability against the wild type was confirmed by two-sided Student’s *t*-test. For all hypotheses, a significance level of 0.05 was used.

### Reporting summary

Further information on research design is available in the Nature Research Reporting Summary linked to this article.

## Supplementary information

Supplementary Information

Description of Additional Supplementary Files

Supplementary Data 1–10

Reporting Summary

## Data Availability

Source data to reproduce the figures and tables of this paper are available at Zenodo (10.5281/zenodo.5775246)^54^. The sequence data are deposited in the DDBJ/EMBL/Genbank nucleotide sequence database under accession numbers DRA011959 and AP018823.

## Code availability

Analytical codes and input data files are publicly available at Zenodo (10.5281/zenodo.5775246)^54^.
