# Supplementary material for: Genome-wide identification of bacterial colonization and fitness determinants on the floating macrophyte, duckweed
Source: Commun Biol. 2022 Jan 19;5:68. doi: 10.1038/s42003-022-03014-7 (PMC8770550; doi:10.1038/s42003-022-03014-7)
Supplement: Supplementary file 1 — Supplementary Information [file 42003_2022_3014_MOESM1_ESM.pdf]

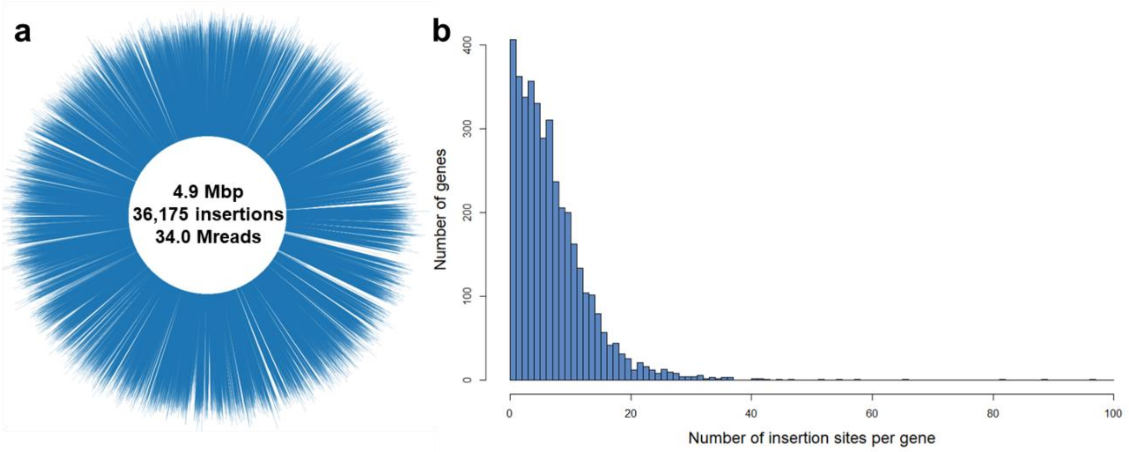

Fig. S1. Properties of transposon insertion mutant library. (A) Genome-wide distribution of insertion sites in the mutant library. Chromosomal position and $\log_{10}$ lead counts of insertion sites are shown. Blue and grey bars represent insertions in cording sequences and non-coding sequences, respectively. (B) A histogram showing the number of insertion sites per gene.

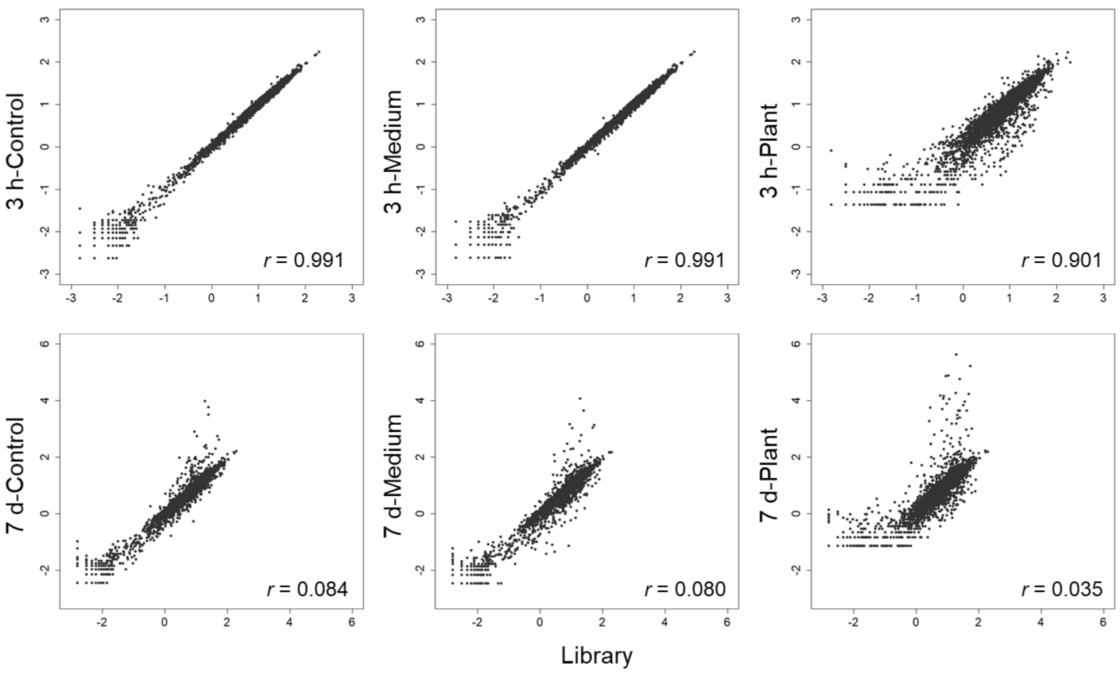

Fig. S2. Comparison of gene detection frequencies with inoculated mutant library. Axes indicate the Log_{10} gene-level detection frequency.

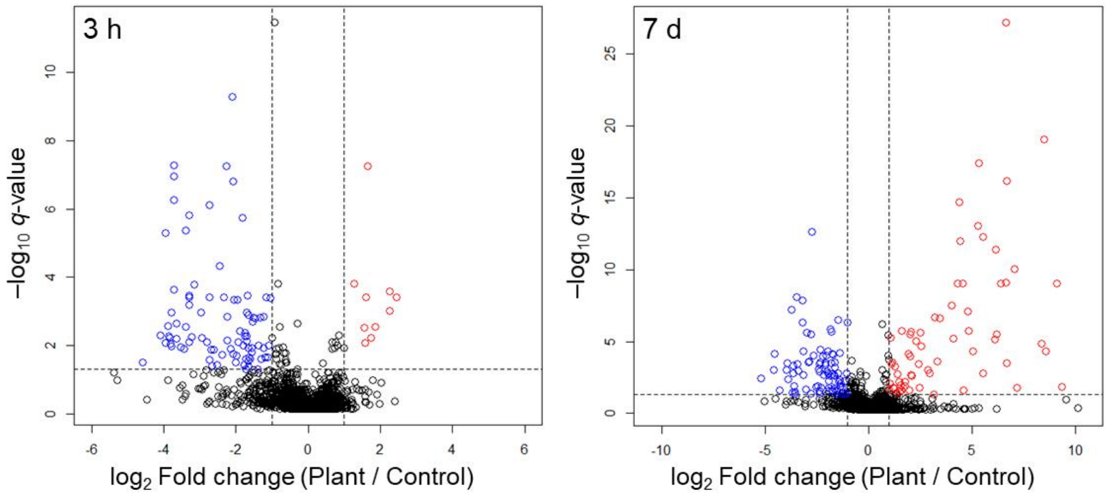

Fig. S3. Determination of depleted and enriched genes of *Aquitalea magnusonii* H3. We determined depleted genes (blue plots) and enriched genes (red plots) based on effect size (> 2-fold) and significance ($q < 0.05$) between Plant and Control samples. Break lines represent threshold values.

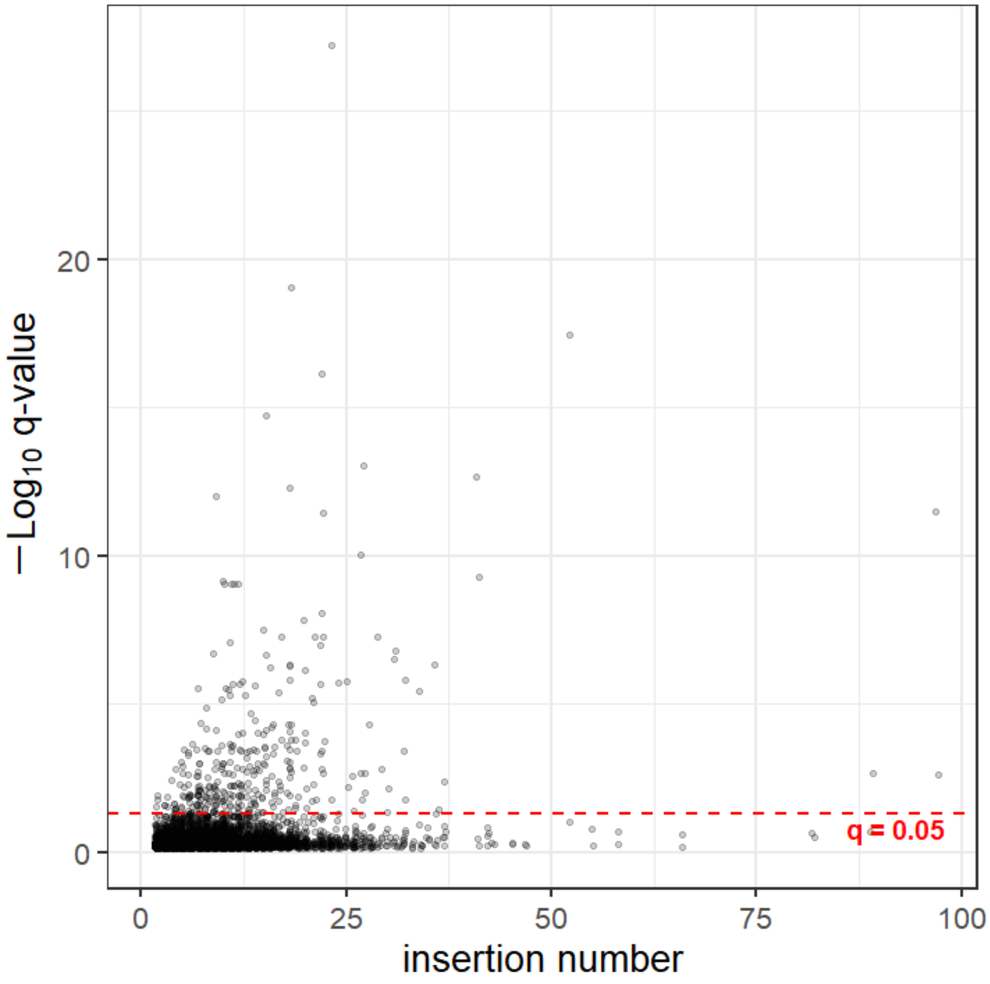

Fig. S4. Relationship between number of insertion sites and statistical power to detect depleted/enriched genes. Each plot represents tested genes (insertion number ≥ 2), and obtained *q*-value was plotted against insertion number. Results of 3-h and 7-d experiments were shown in the same figure (*n* = 6,724 genes).

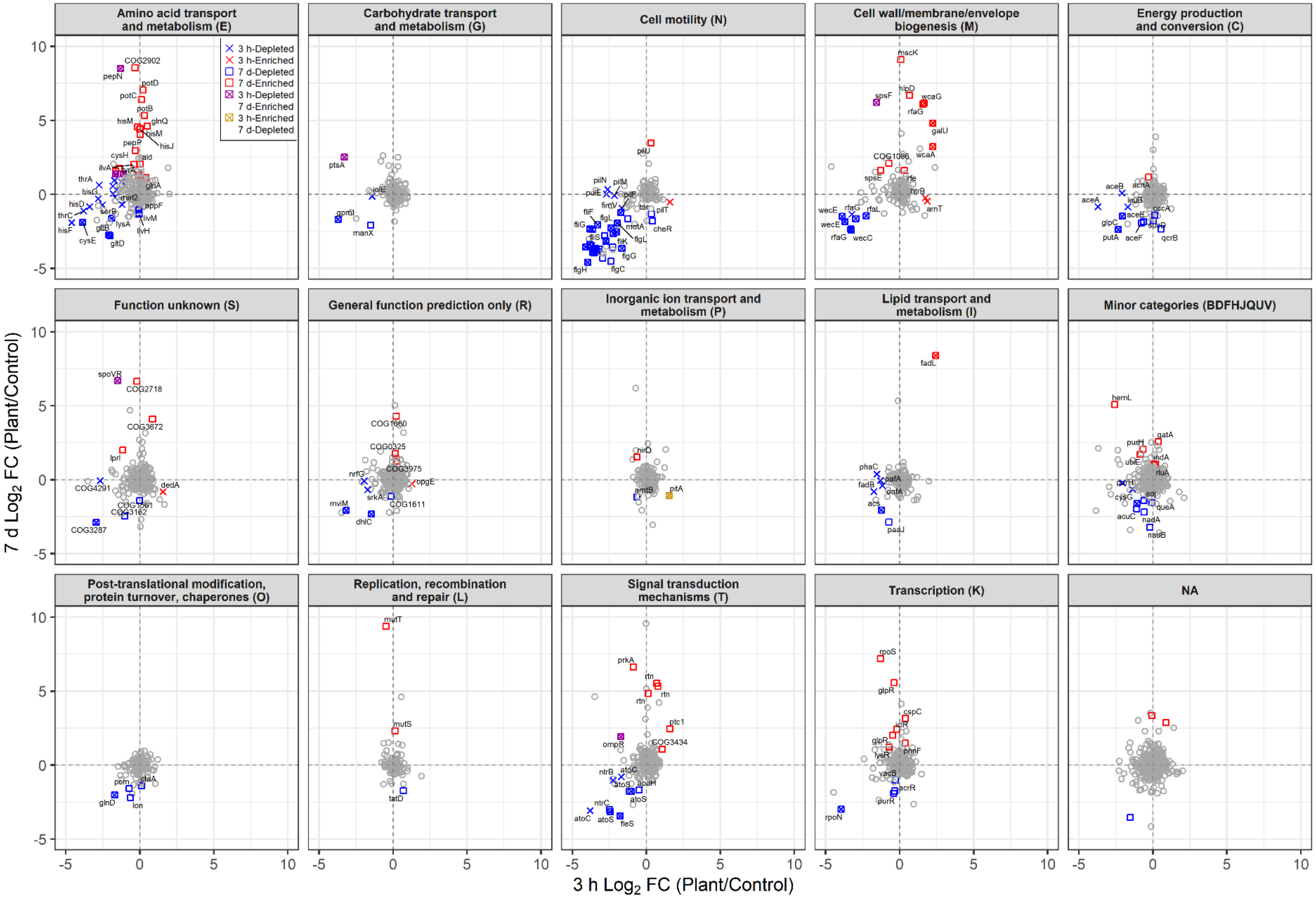

Fig. S5. Comparison of the screening results of 3-h and 7-d experiments. The same plots as Fig. 1d are shown per functional category.

Table S1 COG classification of depleted and enriched genes detected in the Tn-seq. Asterisks indicate the results of enrichment analysis (Fisher's exact test).

COG category	Number of genes >2 insertion sites	3 h		7 d	
		Depleted	Enriched	Depleted	Enriched
Amino acid transport and metabolism (E)	350	21***	0	7	19***
Transcription (K)	245	2	0	5	7
Signal transduction mechanisms (T)	221	7	0	6	7
Energy production and conversion (C)	192	5	0	7	1
Cell wall/membrane/envelope biogenesis (M)	176	8	5***	6	10**
Carbohydrate transport and metabolism (G)	169	3	0	2	1
Inorganic ion transport and metabolism (P)	165	0	1	2	1
Cell motility (N)	142	24***	1	28***	1
Lipid transport and metabolism (I)	130	5	1	2	1
Posttranslational modification, protein turnover, chaperones (O)	117	1	0	4	0
Replication, recombination and repair (L)	103	0	0	1	2
Coenzyme transport and metabolism (H)	99	1	0	3	2
Other categories	913	8	2	9	11
Unclassified to COG	537	0	0	1	2

*, $p < 0.05$; **, $p < 0.01$; ***, $p < 0.001$
