# Supplementary material for: Genome-wide identification of bacterial colonization and fitness determinants on the floating macrophyte, duckweed
Source: Commun Biol. 2022 Jan 19;5:68. doi: 10.1038/s42003-022-03014-7 (PMC8770550; doi:10.1038/s42003-022-03014-7)
Supplement: Supplementary file 2 — Description of Additional Supplementary Files [file 42003_2022_3014_MOESM2_ESM.pdf]

Description of Additional Supplementary Files

File name: Supplementary Data 1
Description: Blast search results against database of essential genes (DEG).

File name: Supplementary Data 2
Description: Tn-seq screening results. Column A-G: Annotation results of strain H3 genes and insertion number in the Tn-seq. Column H-N: Gene-level detection frequency of each gene. Column O-AF: fold change and statistical significance among compared samples.

File name: Supplementary Data 3
Description: A list of depleted genes identified in the 3-h experiment. Gene annotation results and the comparison of gene-level detection frequency among 3 h-Plant and 3 h-Control samples are shown.

File name: Supplementary Data 4
Description: A list of enriched genes identified in the 3-h experiment. Gene annotation results and the comparison of gene-level detection frequency among 3 h-Plant and 3 h-Control samples are shown.

File name: Supplementary Data 5
Description: A list of depleted genes identified in the 7-d experiment. Gene annotation results and the comparison of gene-level detection frequency among 7 d-Plant and 7 d-Control samples are shown.

File name: Supplementary Data 6
Description: A list of enriched genes identified in the 7-d experiment. Gene annotation results and the comparison of gene-level detection frequency among 7 d-Plant and 7 d-Control samples are shown.

File name: Supplementary Data 7
Description: A list of genes in the four chemotaxis gene cluster.

File name: Supplementary Data 8
Description: A list of genes shown in Figure 3.

File name: Supplementary Data 9
Description: A list of genes shown in Figure 4.

File name: Supplementary Data 10
Description: Bacterial strains, vectors, and primers used in this study.
