# Supplementary material for: Genome-wide identification of bacterial colonization and fitness determinants on the floating macrophyte, duckweed
Source: Commun Biol. 2022 Jan 19;5:68. doi: 10.1038/s42003-022-03014-7 (PMC8770550; doi:10.1038/s42003-022-03014-7)
Supplement: Supplementary file 4 — Reporting Summary [file 42003_2022_3014_MOESM4_ESM.pdf]

nature research

Corresponding author(s): Hidehiro Ishizawa

Last updated by author(s): Dec 13, 2021

Reporting Summary

Nature Research wishes to improve the reproducibility of the work that we publish. This form provides structure for consistency and transparency in reporting. For further information on Nature Research policies, see our Editorial Policies and the Editorial Policy Checklist.

Statistics

For all statistical analyses, confirm that the following items are present in the figure legend, table legend, main text, or Methods section.

n/a	Confirmed	
☐	☒	The exact sample size (n) for each experimental group/condition, given as a discrete number and unit of measurement
☐	☒	A statement on whether measurements were taken from distinct samples or whether the same sample was measured repeatedly
☐	☒	The statistical test(s) used AND whether they are one- or two-sided<br>*Only common tests should be described solely by name; describe more complex techniques in the Methods section.*
☒	☐	A description of all covariates tested
☐	☒	A description of any assumptions or corrections, such as tests of normality and adjustment for multiple comparisons
☐	☒	A full description of the statistical parameters including central tendency (e.g. means) or other basic estimates (e.g. regression coefficient) AND variation (e.g. standard deviation) or associated estimates of uncertainty (e.g. confidence intervals)
☐	☒	For null hypothesis testing, the test statistic (e.g. F, t, r) with confidence intervals, effect sizes, degrees of freedom and P value noted<br>*Give P values as exact values whenever suitable.*
☒	☐	For Bayesian analysis, information on the choice of priors and Markov chain Monte Carlo settings
☒	☐	For hierarchical and complex designs, identification of the appropriate level for tests and full reporting of outcomes
☐	☒	Estimates of effect sizes (e.g. Cohen's d, Pearson's r), indicating how they were calculated

Our web collection on statistics for biologists contains articles on many of the points above.

Software and code

Policy information about availability of computer code

Data collection	Measurement of colony diameter; imageJ v1.51n
Data analysis	Quality filter of sequencing data; prinseq v0.20.4<br>Mapping of Tn-seq reads; pyinseq v0.2.0<br>Statistical analyses; R v3.6.2

For manuscripts utilizing custom algorithms or software that are central to the research but not yet described in published literature, software must be made available to editors and reviewers. We strongly encourage code deposition in a community repository (e.g. GitHub). See the Nature Research guidelines for submitting code & software for further information.

Data

Policy information about availability of data

All manuscripts must include a data availability statement. This statement should provide the following information, where applicable:
- Accession codes, unique identifiers, or web links for publicly available datasets
- A list of figures that have associated raw data
- A description of any restrictions on data availability

Analytical codes and input data files are publicly available on Zenodo (https://doi.org/10.5281/zenodo.5775246). The sequence data that support the findings of this study are deposited in the DDBJ/EMBL/Genbank nucleotide sequence database under accession number DRA011959 and AP018823.

Field-specific reporting

Please select the one below that is the best fit for your research. If you are not sure, read the appropriate sections before making your selection.

☒ Life sciences ☐ Behavioural & social sciences ☐ Ecological, evolutionary & environmental sciences

For a reference copy of the document with all sections, see nature.com/documents/nr-reporting-summary-flat.pdf

Life sciences study design

All studies must disclose on these points even when the disclosure is negative.

Sample size	Tn-seq; number of different mutants of the same gene are considered as replicates. The number is known after sequencing and analysis.<br>Co-colonization assay; n = 3, following to conventional methods of similar experiments.<br>Motility assay; n = 6, following to conventional methods of similar experiments.<br>qPCR; n = 3, following to conventional methods of similar experiments.
Data exclusions	No data was excluded from analyses except for low-quality sequence reads.
Replication	Tn-seq experiment was done once and the results were confirmed by the follow-up experiments. Experiments other than Tn-seq were repeated equal or more than twice; in all cases yielded similar outcomes as shown in the paper.
Randomization	Not applicable.
Blinding	There was no blinding. Since the data was obtained by very digital processes (sequencing or counting colonies of bacteria), we do not expect that a bias is introduced by the investigator.

Reporting for specific materials, systems and methods

We require information from authors about some types of materials, experimental systems and methods used in many studies. Here, indicate whether each material, system or method listed is relevant to your study. If you are not sure if a list item applies to your research, read the appropriate section before selecting a response.

Materials & experimental systems

n/a	Involved in the study	
☒	☐	Antibodies
☒	☐	Eukaryotic cell lines
☒	☐	Palaeontology and archaeology
☒	☐	Animals and other organisms
☒	☐	Human research participants
☒	☐	Clinical data
☒	☐	Dual use research of concern

Methods

n/a	Involved in the study	
☒	☐	ChIP-seq
☒	☐	Flow cytometry
☒	☐	MRI-based neuroimaging
